# Distorted body weight perception and its gender differences in middle-aged adults: population based study

**DOI:** 10.1186/s12889-020-8358-9

**Published:** 2020-03-02

**Authors:** Youngshin Song, Myoungjin Kwon, Sun Ae Kim

**Affiliations:** 1 College of Nursing, Chungnam National University, Jung-gu, Munhwa-ro 266, Daejeon, 35015 Republic of Korea; 2 Department of Nursing, Daejeon University, 62 Daehak-ro, Dong-gu, Daejeon, 34520 Republic of Korea; 3 Department of Nursing, Korea National University of Transportation, 61 Daehak-ro, Yonggang-ri, Jeungpyeong-eup, Jeungpyeong-gun, Chungcheongbuk-do 27909 Republic of Korea

**Keywords:** Body image regarding body weight, Perceptual distortion, Middle aged, Body weight, Sex

## Abstract

**Background:**

Increasing interest in appearance and the growing preference for a beautiful body can lead to physical and psychological problems due to an inappropriate body image perception. As such, there is a need to identify what factors may contribute to an inappropriate body image. The purpose of this study was to examine the presence of distorted body weight perception among middle-aged Koreans and identify gender differences and other factors that contribute to a distorted body image regarding body weight.

**Methods:**

Data on 8363 middle-aged adults (aged 45–64 years) from the Korea National Health and Nutrition Examination Survey were analyzed using complex samples analysis considering weight, stratification variables, and cluster variables. The difference between perceived body image regarding body weight and actual body mass index was used to assess distorted body weight perception. Socioeconomic status, health behaviors, daily energy consumption, and psychological status (depression and stress) were assessed for their relationship to distorted body weight perception.

**Results:**

Results showed that a distorted body image regarding body weight was more prevalent among middle-aged men (45.3%) than women (25.7%). Age, income, perceived health status, and health behaviors were significantly associated with distorted body weight perception in middle-aged men, whereas psychological factors were associated with distorted body weight perception in middle-aged women.

**Conclusions:**

Further research on distorted body weight perception is needed to gain understanding of the gender differences between middle-aged men and women in Korea. Furthermore, to the results of the study can be used as a basis for developing various education, health mediation, and public health promotion interventions and programs to address body weight perception in middle-aged adults.

## Background

Body image regarding body weight includes the person’s thoughts and feelings about one’s body, accompanied by subjective perception [1], and is related to one’s physical health and psychosocial well-being [2]. The term “body weight perception” has been used in the same way as body image regarding body weight to describe how perception of one’s body can differ from one’s actual body weight [1, 3]; that is, body weight perception is based on the recognition or awareness of one’s body weight status as normal, underweight, or overweight [4].

Body weight perception can vary according to biological factors such as age and gender [1, 3, 5], sociocultural background [6, 7], and psychological status [8, 9]. Particularly, gender differences regarding body weight perception have been found at certain ages. For example, adolescent boys tend to desire muscularity in terms of their physical appearance, whereas among most girls thinness is perceived as the female body ideal [5, 10]. However, body weight perception can change with age as people become conscious of their declining physical attraction [9, 11]. Generally, in middle age there is relatively less importance placed on physical appearance of the body and there is more concern with one’s self-esteem [11]. For example, in a study of middle-aged Korean women the participants had a negative reaction to their realistic body, which was linked with stress and depression during menopause in women in their 50s [9, 12]. However, in a study of Latin American middle-aged women who experienced body dissatisfaction, their body weight perception was associated with being underweight, employment status, and level of physical activity [13]. As such, body image regarding body weight and related factors varied in women from different countries and places.

With regard to research on men, middle-aged men who compared their appearance to other men tended to be highly concerned about how they looked, and body image disturbance regarding weight negatively affected social and sexual functioning in their interpersonal relationships [10]. Middle-aged men who had such concerns tended to try to hide their body and had low self-esteem [10, 11]. Thus, previous studies that have included middle-aged men tended to emphasize the social aspects of body image related to their body weight [9–11]. Only one study included middle-aged Korean men, and there was no significant gender difference in body image [9]. Prior studies did not have gender differences as the focus of their investigation, in particular with regard to having a distorted body image in middle-aged adults. Body weight perception is present throughout the lifespan, and prior research has focused on women and young adults. Less is known about men and middle-aged adults, or how men and women compare on factors relevant to body weight perception. Thus, gender differences in body weight perception need to be identified. Changes in body image regarding body weight in one’s life is climacteric; therefore, healthcare professionals need to have an understanding of the causes and effects of body perception in middle-aged adults [14].

Before the 1980s when Korea was an underdeveloped country, being overweight was a symbol of wealth and “a sign of plenty.” Even now, older-aged Koreans view being overweight as the ideal body image regarding body weight, whereas young Koreans apparently want to be thin. However, recently there has been a growing tendency to place importance on appearance in Korea. Middle-aged men and women are also pursuing thinness under the influence of urbanization and the mass media, and this trend is causing side effects such as poor eating habits and inadequate consumption of health supplements. As mentioned before, inappropriate body weight perception sometimes can be associated with psychological problems such as depression. Therefore, recognizing the importance of public health, the Korean government conducts a health and nutrition survey every two years that includes the assessment of body weight perception.

With the rapid change of traditional Korean beliefs related to body image regarding body weight and limited research on middle-aged men and women, the aim of the present study was exploratory with a focus on distorted body image regarding body weight in Korean men and women.

Our research questions were as follows: Is there a difference between middle-aged men and women in body image regarding body weight distortion? If so, do middle-aged men and women have different factors related to their distorted body image regarding body weight? The answers to these questions can increase our understanding of weight-related health behaviors in Korean middle-aged adults.

## Methods

### Study design

The present study was a cross-sectional descriptive study that was conducted nationwide to identify differences in distorted body weight perception and its related factors according to gender among middle-aged Koreans.

### Data source and participants

Data for this study were obtained from the Korea National Health and Nutrition Examination Survey (KNHANES). The KNHANES has been administered annually by the Korea Centers for Disease Control and Prevention (KCDC) since 1998 using a multi-stage clustered probability sampling method. The participants in the KNHANES are a nationally representative sample of civilians in Korea, and about 10,000 individuals aged one year and over are included each year. Therefore, the potential for sampling bias in the data can be limited.

The KNHANES consists of three components (health interview, health examination, and nutrition survey) and is performed by trained medical staff at a mobile examination center and dieticians’ visits to the homes of the participants.

In this study, raw data were extracted from the 6th KNHANES (2014–2015) and 7th KNHANES (2016–2017) and collected by the stratified colonies system extraction method. All statistics of this survey have been calculated using sample weights assigned to sample participants. Among all data (*N* = 31,207), we extracted data on the target population of this study, which was middle-aged adults (45–64 years old) (*N* = 9172). We excluded participants who had missing data on body weight perception and BMI (*N* = 809), resulting in a final weighted sample of 8363 (91.17% of the total middle-aged sample).

### Assessment of measurements

Among the three components of the KNHANES, socioeconomic status, anthropometric measures, body weight perception, health behaviors, food intake, and psychological status (perceived stress, depression, and suicidal ideation) were measured.

#### Socioeconomic status

Age, education level (≤ middle school versus > middle school), having a spouse (yes or no), having a job (yes or no), type of job (blue-collar worker or white-collar worker), household income quartile (the lowest, low, moderate, high), number of family members living together, and type of health insurance (National Health Insurance, National Workplace Health Insurance, Medical Aid) were used to assess socioeconomic status.

#### Anthropometric measures

Body mass index (BMI) was calculated using height (cm) and weight (kg) and classified into three groups: less than 18.5 kg/m^2^ = underweight, 18.5–22.9 kg/m^2^ = normal weight, more than 23.0 kg/m^2^ = overweight [12]. One item asked about “weight changes” in the last one year, and responses included “no change,” “weight loss,” and “weight gain.”

Daily energy consumption was classified as insufficient or excessive energy intake according to the Korean Foods and Nutrients Guideline (KFNG) [15]. Participants who consumed less than the recommended amount of daily energy intake were categorized as the insufficient group, and those who consumed more than the recommended amount of daily energy intake were categorized as the excessive group. In the KFNG, the recommended amount of daily energy consumption varies by age: 1900 kcal for women and 2400 kcal for men aged 30–49 years, and 1800 kcal for women and 2200 kcal for men aged 50–64 years [14].

#### Body weight perception

To assess body weight perception, participants were asked “what do you think about your body weight?” Response choices included “perceived as thinner,” “perceived as normal,” and “perceived as obese.” Distorted body image regarding body weight was measured by comparing the difference between perceived body image regarding body weight and actual body size based on BMI categories [1]. Consequently, participants were categorized into two groups: the non-distorted group, in which perceived body weight corresponded with the BMI category; and the distorted group, in which perceived body weight was below their BMI category (underestimation) or was above their BMI category (overestimation).

#### Health behaviors

Health behaviors consisted of regular participation in the national health screening program (yes or no), perceived health status (good or not bad/bad), having daily activity limitations (yes or no), weight control effort (yes or no), drinking (yes or no), heavy drinker (yes or no), smoking (yes or no), and regular daily walking exercise (yes or no). Heavy drinker was considered drinking on average 40 g/day (pure alcohol) or more in men and 20 g/day or more in women. Because there is no official guideline on a single standard drink size in Korea, we defined the amount of alcohol per 1 standard drink as 8 g in accordance with the guidelines on alcohol consumption [16]. For women we also gathered data regarding menopause (with or without).

#### Psychological status

Participants were asked about feelings of depression lasting more than two weeks, any suicidal ideation in the past year, and the presence of stress in daily life. Each question was responded to with “Yes” or “No.”

### Statistical analysis and complex samples analysis

To increase the sensitivity and normality of this study, and reduce sampling bias, the complex samples analysis procedures were implemented by considering sampling weights, stratification variables, and cluster variables using the IBM SPSS 25.0 program.

Most statistical analyses assume that the data collected are from a simple random sample of the population of interest; however, if sampling units are widely spread out geographically, complex sampling designs are more efficient than simple random samples both in the choice of participants and in the feasibility of collecting data.

The Korea Centers for Disease Control and Prevention recommends complex sample analysis for analysis using raw data from the KNHANES. Complex sample data analysis programs such as SPSS Complex Samples analysis are designed to address the sampling design elements such as weights, stratification, and clusters. Prior to conducting the data analysis, researchers should merge multiple data files to create a complex sample plan file. In this case, weights are used to improve representativeness and accuracy. Weight were given from the KNHANES. This is because there were limitations in extracting data evenly from the population, and non-response errors can also differ due to differences in the number of households and populations between design and survey time. According to KNHANES’ raw data analysis guide, missing data is treated as a “system missing value” in the analysis. Missing data was statistically excluded. First, data from the selected sample (adults aged 45–64) were analyzed separately by gender. Cross tabulation analysis was conducted to identify the distribution of distorted and non-distorted participants in each gender. Next, descriptive analysis was performed to examine the distribution of all variables by gender. Complex samples chi-square tests and t-test were then conducted to compare the percentage or mean of all variables by gender. Finally, variables that had a significant association in the bivariate analyses were entered in a logistic regression model. Logistic regression was performed to examine the association between distorted body weight perception and the variables according to gender. The significance level was set at .05.

## Results

### Body weight perception by gender

The gender distribution of the total sample (*N* = 8363) was 42.5% men (*N* = 3552) and 57.5% women (*N* = 4811). The percentage of men whose perceived weight corresponded with their actual BMI was 32.1%, whereas 69.1% of women correctly estimated their weight. The percentage of men who had a distorted body image regarding body weight was 45.3% (*N* = 1610), while 25.7% (*N* = 1236) of women had a distorted body image regarding body weight (Table 1).

**Table 1:** Distributions of body weight perception and BMI by gender (*N* = 8363)

Gender	Perception	BMI (kg/m^2^)		
		< 18.5, *n* (%)	18.5–22.99, *n* (%)	≥ 23.0, *n* (%)
Men (*n* = 3552)	Underweight	57 (91.9) ^a)^	496 (49.7) ^b)^	80 (3.2) ^b)^
	Normal	4 (6.5) ^b)^	477 (47.8) ^a)^	1005 (40.3) ^b)^
	Overweight	1 (1.6) ^b)^	24 (2.5) ^b)^	1408 (56.5) ^a)^
Total	*n* (%)	62 (100)	997 (100)	2493 (100)
Women (*n* = 4811)	Underweight	93 (92.1) ^a)^	354 (18.4) ^b)^	28 (1.0) ^b)^
	Normal	8 (7.9) ^b)^	1294 (67.3) ^a)^	572 (20.5) ^b)^
	Overweight	0 (0) ^b)^	274 (14.3) ^b)^	2188 (78.5) ^a)^
Total	*n* (%)	101 (100)	1922 (100)	2788 (100)

*BMI* body mass index, ^a)^ non-distorted group, ^b)^ distorted group

Total number of ^a)^ non-distorted: 1942 (54.7%) for men and 3575 (74.3%) for women

Total number of ^b)^ distorted: 1610 (45.3%) for men and 1236 (25.7%) for women

### Body image regarding body weight according to participant characteristics

For men, the distribution of distorted and non-distorted body image regarding body weight was significantly different according to age (*p* < .001), education level (*p* = .001), income quartile (*p* = .007), number of chronic diseases (*p* < .001), perceived health status (*p* < .001), change in body weight (*p* < .001), weight control effort (*p* < .001), heavy drinking (*p* < .001), and regular walking (*p* = .003).

For women, there were significant differences according to age (*p* = .001), education level (*p* = .004), type of job (*p* = .017), number of chronic diseases (*p* = .021), regular cancer screening (*p* = .036), change in body weight (*p* < .001), experiencing stress (*p* = .031), depressive symptoms (*p* < .001), and suicidal ideation (*p* = .002) (Table 2).

**Table 2:** Differences in distorted body image regarding body weight according to characteristics in each gender

		Men (*n* = 3552)				Women (*n* = 4811)			
Characteristics	Classification	*n* (weighted %)/ *M*	Distorted (*n* = 1610) *n* (weighted %)/*M* (*SD*)	Non-distorted (*n* = 1942) *n* (weighted %)/*M* (*SD*)	X^2^/t (sig.)	*n* (weighted %)/*M*	Distorted (*n* = 1236) *n* (weighted %)/*M* (*SD*)	Non-distorted (*n* = 3575) *n* (weighted %)/*M* (*SD*)	X^2^/t (sig.)
Mean age	years	54.67	54.99(5.59)	54.40(5.71)	−4.67 (<.001)	54.53	54.97(5.73)	54.38(5.59)	−3.31 (.001)
Educational level (*n* = 7909)	Middle school and under	938(25.9)	469(28.5)	469(23.8)	9.43 (.001)	1809(36.7)	507(39.9)	1302(35.6)	6.69 (.004)
	High school and over	2386(74.1)	1024(71.5)	1362(76.2)		2776(63.3)	650(60.1)	2126(64.4)	
Having spouse (*n* = 8363)	Yes	3371(94.6)	1529(94.9)	1842(94.4)	0.37 (.552)	4731(98.4)	1215(98.3)	3516(98.4)	0.10 (.670)
	No	181(5.4)	81(5.1)	100(5.6)		80(1.6)	21(1.7)	59(1.6)	
Having job (*n* = 7904)	Yes	2816(85.7)	1269(86.5)	1547(85.2)	1.14 (.245)	2702(58.6)	694(58.9)	2008(58.5)	0.06 (.743)
	No	504(14.3)	223(13.5)	281(14.8)		1882(41.1)	462(41.1)	1420(41.5)	
Type of job (*n* = 5518)	White color	990(35.7)	419(34.4)	571(36.8)	1.70 (.114)	699(25.7)	156(22.8)	543(26.7)	4.27 (.017)
	Blue color	1826(64.3)	850(65.6)	976(63.2)		2003(74.3)	538(77.2)	1465(73.3)	
Income quintile (*n* = 8338)	The lowest	877(24.8)	423(26.2)	454(23.6)	8.37 (.007)	1185(24.1)	320(25.9)	865(23.4)	4.28 (.068)
	Low	897(25.2)	434(26.5)	463(24.2)		1194(24.5)	318(25.0)	876(24.3)	
	Moderate	869(24.5)	372(23.2)	497(25.5)		1212(25.6)	301(24.2)	911(26.0)	
	High	897(25.5)	376(24.1)	521(26.7)		1207(25.8)	295(24.8)	912(26.2)	
Number of family members (*n* = 8363)	1	321(8.3)	156(8.9)	165(7.9)	3.56 (.055)	409(6.6)	102(6.4)	307(6.7)	0.18 (.845)
	2–3	1991(53.0)	928(54.1)	1063(52.2)		3097(64.0)	798(64.4)	2299(63.8)	
	4 over	1240(38.6)	526(37.0)	714(39.9)		1305(29.4)	336(29.2)	969(29.5)	
Health security (*n* = 8316)	National regional health insurance	1244(35.3)	571(35.0)	673(35.5)	2.65 (.113)	1598(33.2)	430(34.5)	1168(32.8)	3.54 (.057)
	National workplace health insurance	2137(60.6)	954(60.3)	1183(60.8)		3013(63.3)	756(62.7)	2257(63.5)	
	Medical aid	154(4.1)	75(4.7)	79(3.7)		170(3.5)	37(2.8)	133(3.8)	
Characteristics	Classification	*n* (weighted %)/*M*	Distorted (*n* = 1610) *n* (weighted %)/*M* (*SD*)	Non-distorted (*n* = 1942) *n* (weighted %)/*M* (*SD*)	X^2^/t (sig.)	*n* (weighted %)/*M*	Distorted (*n* = 1236) *n* (weighted %)/*M* (*SD*)	Non-distorted (*n* = 3575) *n* (weighted %)/*M* (*SD*)	X^2^/t (sig.)
Number of chronic diseases.		0.48	0.39(0.63)	0.55(0.73)	11.11 (<.001)	0.49	0.45(0.66)	0.49(0.71)	2.36 (.021)
Regular health screening (*n* = 7922)	Yes	2477(73.6)	1089(72.5)	1388(74.5)	1.66 (.137)	3497(75.0)	896(76.3)	2601(74.5)	1.47 (.130)
	No	853(26.4)	408(27.5)	445(25.5)		1095(25.0)	262(23.7)	833(25.5)	
Regular cancer screening (*n* = 7920)	Yes	2175(63.3)	955(62.6)	1220(63.9)	0.55 (.374)	3455(74.1)	886(75.9)	2569(73.5)	2.53 (.036)
	No	1155(36.7)	541(37.4)	614(36.1)		1137(25.9)	272(24.1)	865(26.5)	
Perceived health status (*n* = 7949)	Good	1056(32.3)	508(35.8)	548(29.5)	14.82 (<.001)	1092(23.6)	266(23.0)	826(23.9)	0.32 (.531)
	Bad or not bad	2284(67.7)	991(64.2)	1293(705)		3517(76.4)	898(77.0)	2619(76.1)	
Daily activity limitation (*n* = 7927)	Yes	261(7.4)	117(7.2)	144(7.6)	0.16 (.608)	380(8.0)	95(7.2)	285(8.3)	1.56 (.090)
	No	3072(92.6)	1381(92.8)	1691(92.4)		4214(92.0)	1064(92.8)	3150(91.7)	
BMI, kg/m^2^ (*n* = 8363)	Less than 18.5	62(1.5)	5(0.1)	57(2.6)	64.25 (<.001)	101(2.2)	8(0.8)	93(2.6)	101.02 (<.001)
	18.5–22.9	997(28.8)	520(32.5)	477(25.8)		1922(41.1)	628(52.8)	1294(37.1)	
	More than 23.0	2493(69.7)	1085(67.4)	1408(71.6)		2788(56.7)	600(46.3)	2188(60.3)	
Body weight perception (*n* = 8363)	Underweight	633(17.3)	576(35.6)	57(2.6)	2340.15 (<.001)	475(9.7)	382(30.5)	93(2.6)	893.84 (<.001)
	Normal	1486(42.2)	1009(62.8)	477(25.8)		1874(39.2)	580(45.2)	1294(37.1)	
	Overweight	1433(40.5)	25(1.7)	1408(71.6)		2462(51.1)	274(24.3)	2188(60.3)	
Change in body weight (*n* = 8359)	No change	2601(72.8)	1223(75.4)	1378(70.7)	34.44 (<.001)	3046(62.7)	865(68.4)	2181(60.7)	73.21 (<.001)
	Weight loss	491(14.3)	238(15.3)	253(13.5)		507(10.4)	167(13.5)	340(9.4)	
	Weight gain	459(12.9)	148(9.3)	311(15.8)		1255(26.9)	202(18.1)	1053(29.8)	
Weight control effort (*n* = 8361)	Yes	2242(63.2)	911(57.6)	1331(67.7)	38.53 (<.001)	3539(74.5)	876(72.7)	2663(75.1)	2.59 (.074)
	No	1310(36.8)	699(42.4)	611(32.3)		1270(25.5)	358(27.3)	912(24.9)	
Menopause (*n* = 4811)	Yes					3241(63.9)	839(64.6)	2402(63.7)	0.31 (.587)
	No					1570(36.1)	397(35.4)	1173(36.3)	
Characteristics	Classification	*n* (weighted %)	Distorted (*n* = 1610) *n* (weighted %)	Non-distorted (*n* = 1942) *n* (weighted %)	X^2^/t (sig.)	*n* (weighted %)	Distorted (*n* = 1236) *n* (weighted %)	Non-distorted (*n* = 3575) *n* (weighted %)	X^2^/t (sig.)
Drinking (*n* = 8362)	Yes	3395(95.8)	1531(65.4)	1864(96.0)	0.92 (.268)	4053(84.9)	1026(83.9)	3027(85.2)	1.28 (.151)
	No	157(4.2)	79(4.6)	78(4.0)		757(15.1)	209(16.1)	548(14.8)	
Heavy drinker (*n* = 6103)	Yes	1831(62.5)	787(59.4)	1044(65.0)	9.73 (<.001)	501(16.9)	135(18.9)	366(16.2)	2.84 (.067)
	No	1141(37.5)	566(40.6)	575(35.0)		2630(83.1)	637(81.1)	1993(83.8)	
Smoking (*n* = 8356)	Yes	2969(83.5)	1332(82.6)	1637(84.3)	1.83 (.108)	391(8.7)	102(8.7)	289(8.7)	0.01 (.983)
	No	581(16.5)	276(17.4)	305(15.7)		4415(91.3)	1131(91.3)	3284(91.3)	
Regular walking (*n* = 5334)	Yes	970(43.3)	440(64.1)	530(41.1)	5.77 (.003)	1544(51.9)	374(50.6)	1170(52.3)	0.63 (.320)
	No	1326(56.7)	590(53.9)	736(58.9)		1494(48.1)	388(49.4)	1106(47.7)	
Energy Consumption (*n* = 7248)	Insufficient	1484(50.3)	667(50.2)	817(50.3)	0.01 (.918)	2713(62.9)	705(64.0)	2008(62.6)	0.73 (.251)
	Excessive	1438(49.7)	659(49.8)	779(49.7)		1613(37.1)	402(36.0)	1211(37.4)	
Stress (*n* = 8353)	Feeling a lot	844(24.3)	368(23.7)	476(24.7)	0.43 (.405)	1205(25.7)	329(27.8)	876(25.0)	3.74 (.031)
	A little feeling	2706(75.7)	1240(76.3)	1466(75.3)		3598(74.3)	902(72.2)	2696(75.0)	
Depressive symptoms (*n* = 4387)	Yes	190(9.9)	91(11.0)	99(9.0)	1.99 (.088)	364(14.7)	109(18.9)	255(13.4)	10.66 (<.001)
	No	1712(90.1)	782(89.0)	930(91.0)		2121(85.3)	507(81.1)	1614(86.6)	
Suicidal ideation (*n* = 4388)	Yes	83(4.3)	34(3.9)	49(4.6)	0.53 (.304)	149(6.3)	49(8.9)	100(5.5)	8.77 (.002)
	No	1820(95.7)	839(96.1)	981(95.4)		2336(93.7)	567(91.1)	1769(94.5)	

*BMI* body mass index

### Distorted body weight perception and its related factors in each gender

The results of the complex samples logistic regression model for each gender are shown in Tables 3 and 4. In men, age was positively associated with a distorted body image regarding body weight (OR = 1.03; 95% CI 1.01–1.04). Additionally, the lowest (OR = 0.68; 95% CI 0.52–0.87) and low income quartile (OR = 0.75; 95% CI 0.60–0.94), fewer chronic diseases (OR = 0.55; 95% CI 0.47–0.63), perceived health status as “bad” or “not-bad” (OR = 1.22; 95% CI 1.04–1.43), no regular walking (OR = 1.33; 95% CI 1.12–1.58), weight gain (OR = 1.48; 95% CI 1.16–1.87), no weight control effort (OR = 0.64; 95% CI 0.53–0.75), and no heavy drinking (OR = 0.81; 95% CI 0.69–0.94) were significantly associated with the presence of distorted body weight perception (Table 3).

**Table 3:** Odds ratios for distorted body image regarding body weight among men (*N* = 3552)

Factors (reference)	OR	95% CI	*p*
Age	1.03	1.01–1.04	.001
Educational level (high school and over)			
Middle school and under	0.89	0.72–1.09	.272
Income quintile (high)			
The lowest	0.68	0.52–0.87	.003
Low	0.75	0.60–0.94	.013
Moderate	0.94	0.75–1.17	.942
Number of chronic diseases	0.55	0.47–0.63	<.001
Perceived health status (good)			
Bad or not bad	1.22	1.04–1.43	.014
Regular walking (yes)			
No	1.33	1.12–1.58	.001
Change in body weight (no change)			
Weight loss	0.82	0.63–1.05	.120
Weight gain	1.48	1.16–1.87	.001
Weight control effort (yes)			
No	0.64	0.53–0.75	<.001
Heavy drinker (yes)			
No	0.81	0.69–0.94	.008

**Table 4:** Odds ratios for distorted body image regarding body weight among women (*N* = 4811)

Factors (reference)	OR	95% CI	*p*
Age	1.01	0.99–1.04	.092
Educational level (high school and over)			
Middle school and under	0.91	0.71–1.16	.468
Types of job (white color)			
Blue color	0.84	0.63–1.12	.242
Change of body weight (no change)			
Weight loss	0.70	0.51–0.97	.033
Weight gain	1.70	1.27–2.29	<.001
Regular cancer screening (yes)			
No	1.04	0.75–1.44	.798
Number of chronic diseases	0.88	0.75–1.04	.152
Stress (yes)			
No	0.85	0.66–1.09	.206
Depressive symptoms (yes)			
No	1.04	0.75–1.44	.798
Suicidal ideation (yes)			
No	1.77	1.01–3.09	.044

In women, weight loss (OR = 0.70; 95% CI 0.51–0.97), weight gain (OR = 1.70; 95% CI 1.27–2.29), and no suicidal ideation (OR = 1.77; 95% CI 1.01–3.09) were significantly associated with the presence of distorted body weight perception (Table 4).

## Discussion

The present study explored distorted body image regarding body weight in middle-aged Korean men and women. Specifically, we were interested in whether there is a difference between middle-aged men and women in distorted body image regarding body weight; and if so, are there different factors related to their distorted body image regarding body weight. The findings from this study showed that distorted body weight perception was more prevalent in men, and its related factors differed by gender.

Previous studies have commonly reported that younger people regardless of gender were more likely than older people to perceive their body as fat [1, 12, 17]. In this regard, the prevalence of body image regarding body weight distortion in the present study was relatively low compared to that of the younger population. For example, the prevalence of body image regarding body weight distortion in female college students aged 18–35 years in Iran was 64.13% [1], while in the present study 25.7% of the middle-aged women had a distorted body image. However, the rate of correct perception in the normal body weight group of middle-aged men was low (49.7%) when compared to middle-aged Mediterranean men (69.8%). Thus, a relatively high percentage of middle-aged Korean men at normal body weight were having distorted perception about their weight, whereas 67.3% of the women in the normal BMI category reported they perceived their body correctly. This finding that more middle-aged men than women had a distorted body image regarding body weight was surprising.

Previous research generally indicates that women are more likely to have a distorted body image regarding body weight than men. For example, a study in Brazil reported 60.6% of women and 43% of men aged 20–59 years had distorted body weight perception [16]. However, because various factors such as socioeconomic status, ethnicity, and nationality can have an influence on body weight perception [6, 18], it is not entirely impossible for men to have a higher rate of distorted body weight perception than women. Furthermore, studies on body image regarding body weight reported that being employed in middle age can influence the awareness of one’s body image regarding body weight in both genders [6, 19]. In the present study, approximately 85% of the men were employed compared with 58% of the women, but middle-aged men with a lower income were more likely to have a distorted body image regarding body weight, unlike women.

As such, the factors associated with a distorted body image regarding body weight differed by gender. In men, distorted body weight perception was associated with older age, lower income, the perception that one’s health is bad, weight gain, no effort at weight control, not taking walks regularly, not being a heavy drinker, and low number of chronic disease compared to men with a non-distorted body image regarding body weight. Most of the characteristics could be considered either health-related factors or social factors because of their relevance to social life. There were no psychological factors such as depression. There is not enough prior research to support these findings, but a study found that a distorted body image regarding body weight in middle-aged men not only related to problematic social functioning but also to depression and anxiety symptoms [10]. The current study was a population-based study using archival data, which put limits on our ability to explore body weight perception and its related factors. Therefore, further research is needed to identify other factors related to body image regarding body weight in middle-aged men.

With regard to the female participants, psychological factors were prominent in women with distorted body weight perception. They were more likely to report experiencing stress, depression, and suicidal ideation compared to women with a non-distorted body image regarding body weight. The results are consistent with previous research [7–9, 20], in which emotional status such as depression was found more in women with a distorted body image regarding body weight. Previous studies have explained this result as a menopause-related phenomena. Middle age is a time when women experience menopause-related emotional and physical symptoms [8], and body changes in appearance and functions that menopausal women face can affect the way they perceive their body.

Body weight perceptions were related to attempts at weight control in younger people [12], but in our sample of middle-aged men and women in the distorted body image regarding body weight group there was little effort to change their weight and no real change in their weight. In the men, this trend was also shown in their lifestyle with regard to drinking alcohol and taking regular walks. There were more heavy drinkers and those who did not take a walk regularly in the distorted body image regarding body weight group. One’s perception of one’s body is an important factor related to eating habits and lifestyle behaviors regardless of age [6], which can have an effect on one’s health status. In a study of Korean older women, the more distorted the body image regarding body weight, the higher was the prevalence of obesity [12].

Age has been reported as being the factor that is most strongly associated with distorted body image regarding body weight [3, 21, 22], and body image regarding body weight in middle-aged adults differs by gender due to differences in physical, psychosocial, and cultural backgrounds. However, it remains unclear as to why the distorted body weight perceptions of Korean middle-aged men were affected more by health-related and social factors than the distorted body image regarding body weight of middle-aged women. Using this population-based data set, other possible factors associated with body weight perception could not be identified given the limitations of the data set. Moreover, a precise measurement of body weight perception was not used, so it was difficult to achieve age-specific information in terms of the psychosocial effects on body image regarding body weight. For middle-aged men and women, an in-depth study is needed on what body image regarding body weight means and how this meaning changes as one ages. Qualitative research that subjectively explores the experiences of Korean men and women could provide such answers.

Despite these limitations, this study identified differences in the prevalence of distorted body weight perception between middle-aged men and women, showing that a higher proportion of middle-aged men had a distorted body image regarding body weight. Furthermore, different factors were associated with body image regarding body weight distortion in men compared to women. It is meaningful that the possibility of generalization has increased due to the research findings on representative samples (middle-aged men and women) in Korea. However, interventions need to be developed and evaluated for their effectiveness. The results of this study can be used to inform the development of gender-specific interventions. For example, gender-related factors can be considered when developing and implementing interventions, education programs, and public health promotion to correct distorted body image in middle-aged women and men. Additionally, experimental research can test the effectiveness of gender-related intervention and prevention strategies that promote a healthy body image.

## Conclusion

The prevalence of distorted body weight perception in middle-aged men was higher than in women. Most factors that were associated with distorted body weight perception in men were social and health-related factors, while psychological factors were related to distorted body weight perception in women. Based on this study’s findings, future research needs to explore the meaning of body image regarding body weight in middle-aged men and women and what psychosocial factors cause a change in body image regarding body weight. Additionally, research should examine how the meaning of body image regarding body weight in middle-aged adults is related to their health behaviors.

## Data Availability

Korea National Health and Nutrition Examination Survey data are available in the public domain at (https://knhanes.cdc.go.kr/knhanes/main.do). All data from KNHANES are coded and available freely.

## Abbreviations

BMI: Body Mass Index
KCDC: Korea Centers for Disease Control and Prevention
KFNG: Korean Foods and Nutrients Guideline
KNHANES: Korea National Health and Nutrition Examination Survey
